# Sensorineural hearing loss in Lassa fever: two case reports

**DOI:** 10.1186/1752-1947-3-36

**Published:** 2009-01-29

**Authors:** Peter O Okokhere, Titus S Ibekwe, George O Akpede

**Affiliations:** 1 Department of Medicine, Irrua Specialist Teaching Hospital, Irrua, Nigeria; 2 ENT Division, Department of Surgery, Irrua Specialist Teaching Hospital, Irrua, Nigeria; 3 Department of Pediatrics, Irrua Specialist Teaching Hospital, Irrua, Nigeria

## Abstract

**Introduction:**

Lassa fever is an acute arena viral haemorrhagic fever with varied neurological sequelae. Sensorineural hearing loss is one of the rare complications which occur usually during the convalescent stage of the infection.

**Case presentation:**

The cases of two female patients aged 19 and 43 years old, respectively, with clinical features suggestive of Lassa fever and confirmed by immunoserological/Lassa-virus-specific reverse transcriptase polymerase chain reaction are presented. Both patients developed severe sensorineural hearing loss at acute phases of the infections.

**Conclusion:**

Sensorineural hearing loss from Lassa fever infections can occur in both acute and convalescent stages and is probably induced by an immune response.

## Introduction

Lassa fever is an acute arena viral haemorrhagic fever which was first identified in Lassa village, Borno State in the northeastern region of Nigeria in 1969 [1]. This rodent-borne virus is highly contagious through the urine of *Mastomys natalensis *(the multimammate rat) or via the body fluid of infected humans. The clinical diagnosis is often difficult because of the varied and non-specific modes of presentation of Lassa fever.

Neurological complications, notably sensorineural hearing loss, have been associated with Lassa fever. According to a fairly recent WHO report, deafness occurs in about 25% of Lassa fever patients [2]. However, no case of sensorineural hearing loss from patients confirmed with Lassa fever has been reported in our environment, according to a search we conducted on PubMed. Here, we present the occurence of sensorineural hearing loss in two confirmed cases of Lassa fever in Irrua, Nigeria.

## Case presentation

### Case report 1

A 19-year-old female undergraduate student presented to our hospital on 5 December, 2003, with complaints of high-grade fever of 9 days' duration; vomiting and abdominal pain of 5 days' duration; headache of 4 days' duration; as well as watery, non-mucous, non-bloody diarrhoea and a sore throat, with difficulty in swallowing, of 2 days' duration.

On examination, the patient was acutely ill looking, conscious and alert. Her temperature was 39.4°C; radial pulse was 110 bpm, with regular, normal volume; blood pressure was 100/60 mmHg in the supine position; and the respiratory rate was 22 cycles/minute. A throat examination showed exudative pharyngitis. Petechial haemorrhages, prolonged bleeding from venepuncture sites and nostrils, and bilateral hearing impairment were also noticed. Apart from mild epigastric tenderness, examination of the abdomen, chest- and central nervous systems was unremarkable.

Investigations showed packed cell volume of 33%; erythrocyte sedimentation rate (ESR) of 40 mm/hr (Westergreen); total white blood cell (WBC) count of 2,400 mm; neutrophil count of 30%, lymphocyte count of 70%; proteinuria(++); a throat-swab culture grew alpha haemolytic streptococci; and no malaria parasites were seen on a blood-smear examination.

A clinical diagnosis of probable Lassa fever was made, considering the endemic nature of the disease in our environment. Blood samples were sent for definitive diagnosis. The results were positive for both Lassa virus-specific IgM and Lassa virus-specific reverse transcriptase polymerase chain reaction (RT-PCR), confirming the clinical diagnosis. The patient was admitted and treated with a 10-day course of ribavirin, in addition to other supportive measures. She recovered, but had progressively worsening hearing impairment. She was discharged 11 days after the admission by the physicians and referred to the Ear, Nose and Throat surgeons for management of the worsening hearing disability. A review of our patient showed that the pinnae and external auditory canal appeared normal, while the tympanic membrane was intact and bilaterally shiny. Tuning-fork tests (Rinne and Weber) were equivocal.

A diagnosis of severe sensorineural hearing loss (SNHL) was confirmed with a pure-tone audiogram, revealing a hearing level in the left ear of 70 dB, in the right ear of 75 dB. Four years later, the patient's hearing was still significantly impaired, and currently she uses a hearing-aid.

### Case report 2

A 48-year-old female trader presented to our hospital on 20 August, 2007, with a 2-week history of fever and a 6-day history of bilateral hearing loss. There was no history of use of ototoxic drugs.

Physical examination revealed an ill-looking, conscious and alert woman. The temperature at presentation was 36.9°C; pulse was 110 bpm, regular small volume; blood pressure was 90/50 mmHg (supine position); and respiratory rate was 20 cycles/minute. There was bilateral conjuctival haemorrhage. The pinnae appeared normal, whereas the external auditory meatus contained scanty dry wax which was evacuated manually. The tympanic membranes were also intact and shiny. She was audiologically confirmed to have bilateral severe SNHL (68 and 70 dB hearing levels for left and right ear, respectively), culminating in her communicating through sign language. There was no sign of meningeal irritation. The chest and abdominal examinations were normal.

A clinical diagnosis of probable Lassa fever was made. Laboratory investigations showed a packed cell volume of 34%, total WBC count of 7,300/mm^3^, neutrophil of 82%, lymphocytes of 13% and platelets of 195 000. Urinalysis showed protein (++). Lassa fever virus-specific RT-PCR was positive for Lassa fever, thus confirming the clinical diagnosis (although retrospectively). The patient responded positively to a 10-day course of ribavirin. She was discharged on 4 September, and one year afterwards, severe SNHL still persisted.

## Discussion

Viral infections such as mumps, rubeolla, rubella, herpes zoster and cytomegalo viruses are known causes of hearing loss in human. These infections usually lead to the loss of hair and supporting cells of the cochlear during the active phase of the infection. The tectorial membranes are disrupted and the stria vascularis atrophy leading to end-vessel thrombosis and inner-ear fibrosis. Direct invasion of the spiral ganglion may also result in the loss of integrity of the vestibulocochlear nerve [3]. All these pathogenic processes are known to occur during the acute phase of the viral infections. Often they result in sudden SNHL and, because of limited diagnosis, most are classified as idiopathic. About 57% to 60% of patients are known to recover spontaneously during convalescence [4,5].

In contrast, hearing loss associated with Lassa fever infections usually occurs at the convalescent stage of the infections [6,7]. However, hearing loss following the illness can also occur during the active phase of the illness as illustrated in the above case series. Further deterioration in hearing over time may be recorded even after full recovery, as shown in Case 1. Cummins et al. [7] postulated that the viraemia is not responsible for the hearing loss in Lassa fever infections; instead, such hearing loss is due to immune response reactions against the elements of the inner ear. Similar observations were also made by Liao et al. [8]. This notion is further supported by the fact that early commencement of ribavirin therapy seems not to offer protection against development of SNHL. Recent research has shown that the strain of Lassa viruses found at the West African coast varies in its amino-acid sequence (genome) and tends to elaborate an already exaggerated immune responses, involving high titers of IgG and IgM [9,10]. Consequently, inhabitants of endemic areas of Lassa fever with sub-clinical infections from exposures in the past stand a higher risk of developing SNHL during their lifetime.

One would have expected such an immunologically-induced systemic hearing loss to be bilateral as demonstrated clinically and by audiological assessment in the above case reports. However, reports of unilateral sudden SNHL associated with Lassa fever infection have been documented [11]. This raises further questions on the actual mechanism involved in Lassa fever-induced SNHL. Further research into this mechanism is necessary.

There is no active mode of management for Lassa fever-induced hearing loss. Conservative methods include hyperbaric labyrinthine vasodilators (e.g. nicotinic acid), hyperbaric oxygen and carbogen therapy [12] to enhance the oxygen pressure/perfusion to the inner ear. Steroids and low molecular weight dextran have also been used. However, there is no clear evidence on the efficacy of these methods of treatment. The effect appears to be similar to that seen in the management of idiopathic hearing loss where most improved cases are believed to be spontaneous rather than as a result of treatment [5,13]. Hence, prevention of contact with the Lassa fever virus and its main vector remains the most efficient mode of control. In the endemic areas such as Edo State, Nigeria, where multimammate rats abound [14], total elimination of the vector of the virus is extremely difficult.

Innovative strides towards development of vaccines for Lassa fever infections are currently being made. It was recently reported that trials on primates have been successful [15]. It will be important to determine the possible long-term effects of the vaccines (immunological antibodies) on the inner ear before application to human. This poses further need for research on the mechanism of Lassa virus-induced hearing loss.

## Conclusion

SNHL from Lassa fever infection can occur at both active and convalescent stages and is probably immune induced. However, the definitive pathogenesis is key to advancement into prophylactic and active treatment of the condition. Therefore, further research is urgently needed in this field.

## Abbreviations

SNHL: sensorineural hearing loss; ESR: erythrocyte sedimentation rate; WBC: white blood cell; RT-PCR: reverse transcriptase polymerase chain reaction

## Consent

Written informed consent was obtained from the patients for publication of this case report and accompanying images. A copy of the written consent is available for review by the Editor-in-Chief of this journal.

## Competing interests

The authors declare that they have no competing interests.

## Authors' contributions

TSI was responsible for literature search, part of the research proper and the preparation of manuscript. POO suggested the topic and participated in carrying out the research. GOA supervised the study and edited the manuscript.
